# Anti-angiogenic therapy as potential treatment for adenomyosis

**DOI:** 10.1007/s10456-024-09960-6

**Published:** 2025-01-25

**Authors:** Marissa J. Harmsen, Lynda J. M. Juffermans, Muara O. Kroon, Arjan W. Griffioen, Judith A. F. Huirne

**Affiliations:** 1 Department of Obstetrics and Gynaecology, Amsterdam UMC Location Vrije Universiteit Amsterdam, De Boelelaan 1117, Amsterdam, The Netherlands; 2 Amsterdam Reproduction & Development Research Institute, Amsterdam, the Netherlands; 3 Angiogenesis Laboratory, Department of Medical Oncology, Amsterdam UMC, Cancer Center Amsterdam, Amsterdam, The Netherlands; 4 Cancer Center Amsterdam, De Boelelaan 1117, 1081 HV Amsterdam, The Netherlands

**Keywords:** Adenomyosis, Angiogenesis, Anti-angiogenic agent, CD-1 mice, Tamoxifen, Axitinib

## Abstract

Adenomyosis is characterized by abnormal uterine bleeding, dysmenorrhea and subfertility. Increased expression of angiogenesis markers in adenomyosis presents a treatment opportunity and was studied in an adenomyosis mouse model. Mice were administered tamoxifen (1 mg/kg) on neonatal days 2–5. At six weeks of age, mice received oral treatment with axitinib 3 mg/kg (‘dose I/AX3’, n = 34), axitinib 25 mg/kg (‘dose II/AX25’ n = 34), or with vehicle-only (‘placebo’, n = 34). The prevalence and severity of adenomyosis were assessed. An *adenomyosis severity index* was calculated by multiplying mean grade/mouse by the percentage affected surface area. Angiogenesis-related gene expression was evaluated using real-time quantitative PCR. 101 mice completed adenomyosis induction and could be analyzed. The prevalence of adenomyosis was 30/33 (90.0%) in dose I, 29/34 (85.3%) in dose II, and 30/34 (88.2%) in placebo treated mice (p = 0.78). High grade (2/3) adenomyosis was significantly less prevalent in mice treated with axitinib dose II (n = 19, 55.9%) than in the placebo group (n = 27, 79.4%, p < 0.05). The adenomyosis severity index was reduced by 48% in the axitinib-treated groups (dose I, p < 0.05). The expression of angiogenic growth factors was reduced in the dose I and II axitinib-treated groups compared to the placebo-treated group. Following these promising first results, further research should focus on commonality among different angiostatic drugs, potential side effects, as well as the method and timing of application.

**Supplementary Information:**

The online version contains supplementary material available at 10.1007/s10456-024-09960-6.

## Introduction

Adenomyosis is a benign condition where endometrium tissue is present in the myometrium layer of the uterus[1]. This leads to clinical symptoms of abnormal uterine bleeding, pain, reduced fertility and miscarriages, with a considerable impact on women’s quality of life and subsequently on society[2, 3]. The etiology of adenomyosis has not been fully clarified. Whereas in the past this condition was thought to mainly develop in multiparous women, improved diagnostic methods have shown that it is also present in nulliparous women and it is associated with a 28% lower clinical pregnancy rate, as well as a more than doubled risk of miscarriage in women undergoing IVF[4–6]. Treatment of adenomyosis may potentially prevent progression of the disease to a severe stage and associated reproductive failure. Up to now, we can only treat symptoms of adenomyosis by suppressing the menstrual cycle with hormonal therapy. However, many women experience too many side effects, no full relief of symptoms and have to turn to more radical therapy such as embolization[7] or hysterectomy[8], both are incompatible with the wish to conceive in the future.

Therapy that would target the underlying etiology of adenomyosis might help these patients in a more sustainable manner and with less side effects. Since abnormal uterine bleeding is a cardinal symptom of adenomyosis and there is evidence of both increased expression of angiogenic factors as well as higher vascular density in hysterectomy specimens[9], we hypothesized that angiogenesis might be a key etiological factor and a potential treatment target [9]. Angiogenesis is the arising of new capillaries out of pre-existing blood vessels and occurs in both physiological and pathological processes [10, 11]. It takes place at all stages of the menstrual cycle, with a tendency towards more angiogenesis during the proliferative or follicular phase, when regeneration of the endometrium occurs [12, 13]. It is suggested that increased angiogenesis is one of the underlying mechanisms in the pathophysiology of adenomyosis [9, 14–18]. In adenomyosis, endometrial cells seem to need angiogenesis to invade and establish at an ectopic location [19, 20]. Our group confirmed this in endometrial tissue retrieved from adenomyosis patients and control patients from our clinic that were matched for age, parity, and menstrual phase [21].

Angiogenesis has since long been considered a potential therapeutic target in the treatment of cancer. When the clinical relevance became apparent, its therapeutic potential was also discovered in benign angiogenic diseases, such as age-related macular degeneration and rheumatoid arthritis [22, 23]. Several different angiogenesis regulators and mechanisms were elucidated [24, 25]. In addition, a number of new drugs and vaccines have been developed and are currently being tested in clinical trials [26, 27]. Interestingly, the results of angiogenesis inhibition have been noticeably more effective in benign disease such as macular degeneration than in the treatment of cancer [28]. This triggered our hypothesis that angiogenesis inhibition might prove a promising therapeutic approach in adenomyosis, since it may reduce the growth of ectopic endometrium as well as alleviate symptoms of abnormal uterine bleeding and subfertility. However, in a benign condition such as adenomyosis, less side effects are tolerated than when treating a malignancy. Axitinib is an angiogenesis inhibitor with a high affinity for VEGF receptor 2 (VEGF-R2) and a better toxicity profile [29]. Hypothetically, treating adenomyosis would require a lower dose of angiogenesis inhibitor than the treatment of cancer. The aim of this study is to investigate the therapeutic effect of angiogenesis inhibition in tamoxifen-induced adenomyosis in a murine model.

## Materials and methods

### Preparation of animals

Experiments were approved by the Central Committee for Animal Experiments in the Netherlands. All experiments were performed conform to all relevant regulatory standards. Pregnant CD-1 mice were purchased from Charles River. Each mouse was housed individually during gestation. After giving birth, the dams were housed in one cage with their pups in a controlled environment with 70–80% humidity at 22 °C with a 12 h/12 h light: dark cycle for three weeks. After three weeks, the pups were weaned and allocated to new cages, with a maximum of five animals per cage. Animals were allowed access to chow and water ad libitum. After weaning, the mothers were terminated, the uterus was excised and used as surplus material to optimize immunohistochemical (IHC) staining.

### Mouse model of adenomyosis

**Fig. 1:**
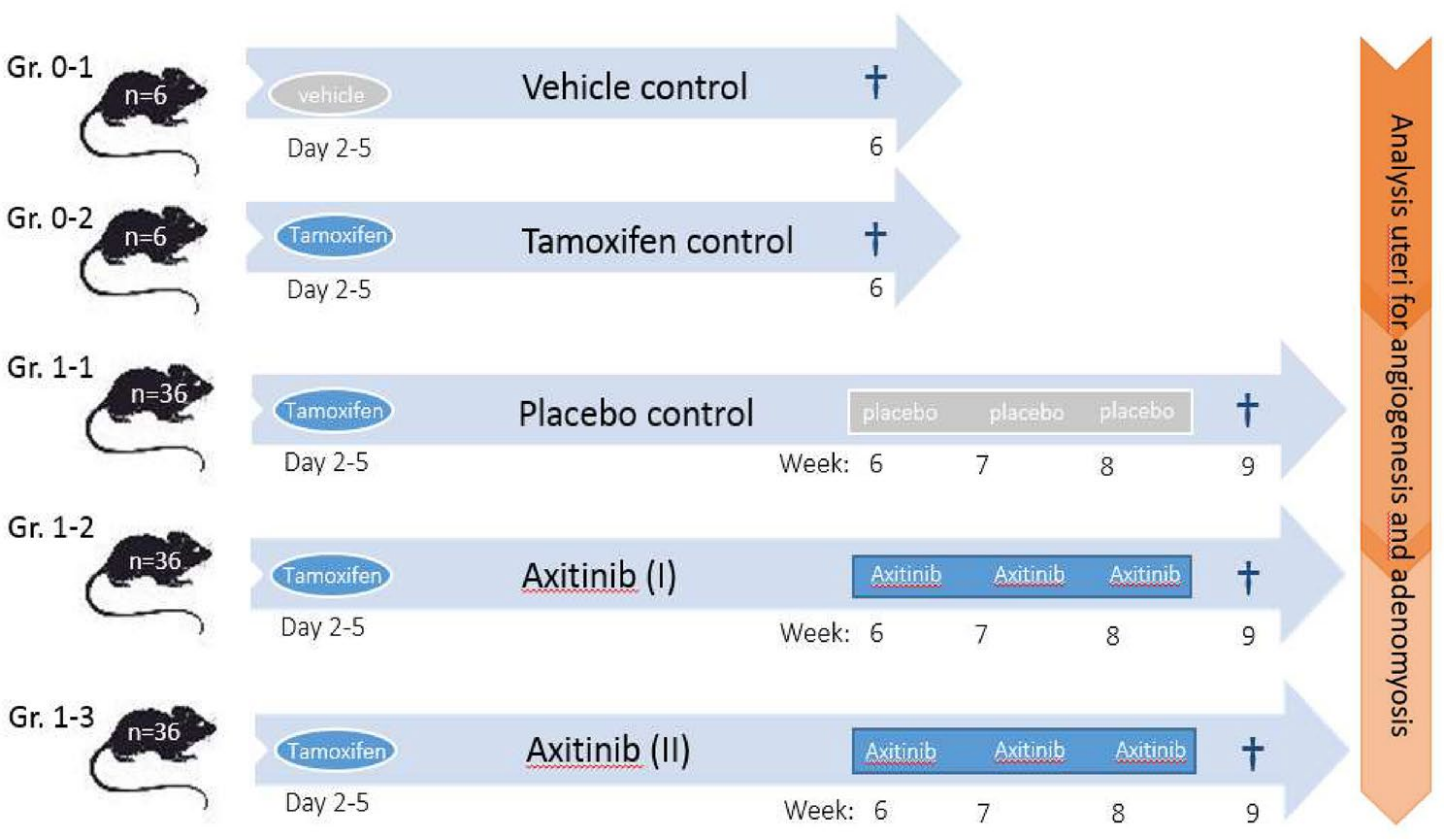
Schematic overview of experimental groups and timeline for inducing adenomyosis in CD1 mice using tamoxifen, followed by treatment with axitinib 3 mg/kg (dose I) or axitinib 25 mg/kg (dose II) from week 6 until week 9. After termination of the mice at week 9, all uteri were analyzed for the presence of adenomyosis

Adenomyosis was induced in the female mice according to a model previously described [30]. To validate this model in our laboratory, female mice were dosed with tamoxifen (1 mg/kg; ‘tamoxifen group’; n = 6), suspended in peanut oil, by oral gavage, at a dose volume of 5 μl/g bodyweight on day 2–5 after birth, as reported previously. [30, 31] As control, female mice (n = 6) received the peanut oil solvent only (‘vehicle group’; n = 6). All mice were sacrificed after six weeks (42 days), to determine the presence and/or degree of adenomyosis. After validation of the model, this model was used to test treatment with anti- VEGFR-2 tyrosine kinase inhibitor axitinib (A-1107; LC Laboratories). To induce adenomyosis, 108 mice received neonatal tamoxifen treatment and were randomly allocated to receive treatment or placebo. On day 42, anti-angiogenesis treatment commenced by oral axitinib treatment or placebo. Axitinib was dissolved in 0.5% sodium carboxymethylcellulose (CMC) at a dose of 3 mg/kg body weight (dose I) or 25 mg/kg body weight (dose II). All animals received treatment by daily oral gavage at 5 µl/g bodyweight with axitinib (dose I/II) or placebo (CMC) for a time period of three weeks. During this period, all animals had daily animal welfare observations, and were weighed at a minimum of three times per week. The experimental groups are depicted in Fig. 1. All mice were sacrificed one day after the last oral gavage day. Blood was drawn, uteri were collected, together with a kidney and liver. The uteri were cut in half, one horn (right/left horn randomized) was fixed in 4% formaldehyde at room temperature for 48 h. Samples were embedded in paraffin for haematoxylin & eosin (H&E) and IHC staining. The other horn was snap-frozen in liquid nitrogen.

### Immunohistochemistry

Uterine tissue was embedded in paraffin and sliced into transverse oriented sections of 5 μm, with three sections of one uterine horn on one microscope slide. Slides were either stained for H&E or used for IHC. Sections were stained for α-smooth muscle actin (α-SMA) (rabbit polyclonal; cat. no. mAb1420; clone 1A4; 1:200; Novus Biologicals), cluster of differentiation 31 (CD31) (rat monoclonal; cat. no. DIA-310 M; clone SZ31; 1:50; Dianova), and vimentin (mouse monoclonal; cat. no. sc-373717; 1:50; Santa Cruz Biotechnology). Paraffin-embedded sections were dewaxed in xylene, rehydrated in 100%, 96%, and 70% ethanol, and washed in PBS (pH 7.4). Antigen retrieval utilizing citrate buffer (pH 6.0) was completed in the autoclave at 98 °C. After washing in PBS, blocking of endogenous peroxidase activity was done by 0.3% H_2_O_2_/PBS for fifteen minutes. For vimentin staining, blocking of endogenous peroxidase was performed before antigen retrieval in the autoclave. After washing in PBS, aspecific binding was blocked by 3% BSA/PBS for one hour. After adding primary antibodies diluted in 0.5% BSA/PBS, sections were incubated overnight at 4 °C. Slides were washed in PBS, subsequently, secondary antibodies were applied for α-SMA (swine anti-rabbit biotinylated antibody; cat. no. ab10935; 1:500; Dako Cytomation), CD31 (donkey anti-rat biotinylated antibody; cat. no. A110-137B; 1:500; Jackson), and vimentin (goat anti-mouse biotinylated antibody; cat. no. ab10721; 1:500; Dako Cytomation) diluted in 0.5% BSA/PBS for 45 min. After washing with PBS, horseradish peroxidase (HRP) – streptavidin (strep) was added (cat. no. ab6734; 1:200; Dako Cytomation) diluted in 0.5% BSA/PBS, for thirty minutes. The sections were then washed in PBS, afterwards 3,3′-Diaminobenzidine (DAB) was added (60 µl DAB; 1 µl 30% H2O2; 1 mL PBS), for eight minutes. Sections were washed in PBS, then counterstained with hematoxylin for thirty seconds. Finally, sections were dehydrated in a mounting absolute alcohol gradient, washed with xylene, and covered with glass coverslips over Quick D mounting medium. Different controls were incorporated by staining a slide without primary antibody, one without primary and secondary antibodies, and one with DAB only.

### Image analysis

Image capture and analysis were performed using an Olympus BX50 microscope and an image capture device. Digital image analysis was performed using ImageFocus (version 4.0) and Image J. All image analyses were performed by two assessors (M.H. and L.J.) who were blinded for the treatment. One to three transverse sections per mouse uterus were analyzed, depending on the quality of the section and/or IHC.

The prevalence and severity of adenomyosis per mouse uterus were assessed by three complimentary methods; (i) the grade of adenomyosis by determining the depth of endometrial gland infiltration on H&E staining, (ii) the percentage of adenomyosis per transverse section by vimentin staining of the ectopic endometrium stroma and (iii) the ratio of thinnest/thickest myometrium by α-SMA immunostaining.

**Fig. 2:**
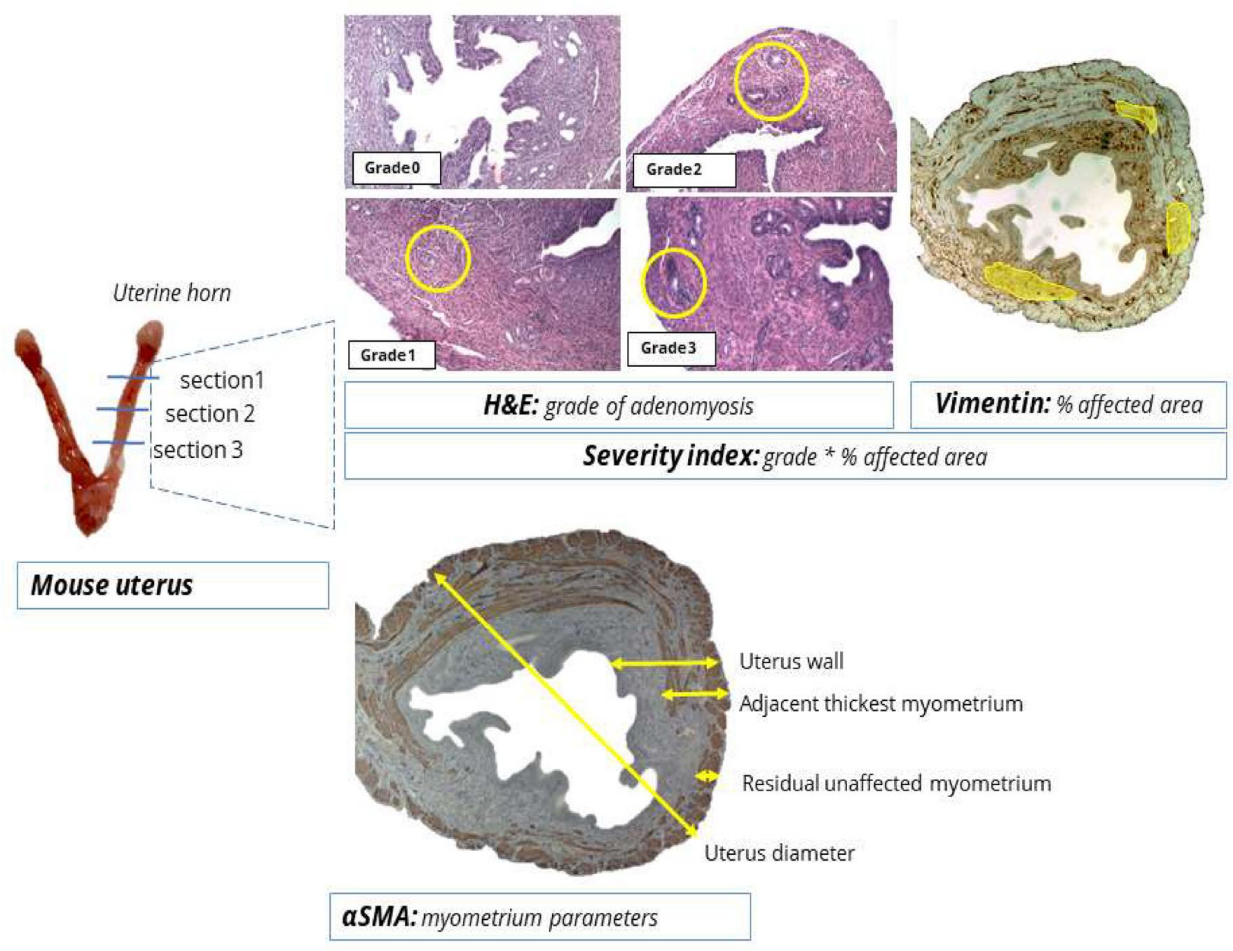
Adenomyosis severity index explained. The grade of adenomyosis (H&E) was multiplied by the surface area of vimentin stained adenomyosis per transverse uterus section. The adenomyosis severity index is the average of all sections analyzed per uterine horn. *Avg.* Average, *H&E* hematoxylin & eosin

Based on H&E staining at 40 × scanning magnification, the diagnosis of adenomyosis was established (yes/no) when endometrial glands were present at ectopic sites in the myometrium, as well as the grade of adenomyosis according to the criteria of Bird et al.[1] as reported previously [32]. Grade 0 was defined as the total absence of ectopic endometrium in the myometrium. Grade 1 was defined as the penetration of ectopic endometrium into superficial myometrium. Grade 2 suggested a penetration of the ectopic endometrium into the middle of the myometrium, and grade 3 was indicated as a penetration past the mid-myometrium. Examples of the classification of depth of ectopic endometrium infiltration are presented in Fig. 2. Both the highest grade per mouse (the deepest infiltration) as well as the mean grade over the analyzed uterine tissue sections per mouse were determined. Additional parameters that were analyzed included the mean number of ectopic endometrium glands, the mean number of ectopic endometrium areas in the myometrium, and the ‘focal’ or ‘diffuse’ presence of adenomyosis. Focal presence was defined as infiltration of ectopic endometrium in only one quartile of the uterus sample. If the presence of ectopic sides was more scattered, it was described as diffuse.

The assessment of the primary outcome of the prevalence and grade of adenomyosis was also analyzed by immunological staining differentiation of the endometrium stroma and the myometrium. Vimentin staining was used to map eutopic and ectopic endometrium stroma and quantify the surface area of ectopic endometrium per uterine section. The visualization of endometrium stroma by vimentin was previously described in endometrium stroma and blood vessels in mice uteri[33]. To estimate the severity of adenomyosis per mouse uterus, a severity index was calculated by multiplying the mean grade of adenomyosis (depth of H&E stained endometrial gland infiltration) times the mean percentage of adenomyosis (vimentin stained ectopic surface area) per mouse uterus. This method is schematically depicted in Fig. 2. Scoring the grade of adenomyosis by of vimentin or α -SMA was performed to validate the grading of the adenomyosis per uterine section. α-SMA immunostaining was used to visualize the possible interruption of the layer of smooth muscle cells of the myometrium and quantify parameters of the uterine wall, including full thickness of the uterine wall (endometrium + myometrium) at thickest point, full thickness of the uterine wall (endometrium + myometrium) at thinnest point, the myometrium at the thinnest point (in adenomyosis affected uteri: residual unaffected myometrium at point of maximum ectopic endometrium infiltration), and the myometrium at the thickest point (in adenomyosis affected uteri: hypertrophied myometrium adjacent to myometrium affected by ectopic). Uterine sections were also examined by experienced pathologists for the presence of fibrosis based on both H&E and phosphotungstic acid haematoxylin (PTAH) staining. Microvascular density (MVD) was quantified by counting CD31 positive blood vessels per unit of measurement in the eutopic endometrium, myometrium, and ectopic endometrium separately. Manual counts were performed in four quadrants of one transversal section per uterine horn, under 400 × magnification.

### RNA isolation, cDNA synthesis and quantitative PCR analysis

To study the efficacy of axitinib treatment at the molecular level, the expression levels of vascular marker CD31 and angiogenic growth factors VEGF-A, VEGF-R1, VEGF-R2, PlGF, and bFGF were measured. RNA was isolated from uterine tissue using an RNA isolation kit from Biorad, according to the supplier’s protocol. Total RNA (1 μg) was reverse transcribed using the iScript reverse transcription supermix for RT-qPCR (Biorad) according to the supplier’s protocol. Following inactivation of the reverse transcriptase activity, copy DNA (cDNA) was stored at − 20 °C. Specific mouse primers were selected and applied as described by Thijssen et al.[34] after synthetization by Sigma-Genosys.

### Statistical analysis

Statistical analysis was performed using Graphpad Prism software version 9.0 (GraphPad Software, San Diego, California, USA) and SPSS 26.0 software (SPSS, Inc.). Pearson Chi-square tests (for trend) were used to compare categorical variables. One-way ANOVA and independent samples T-test for normally distributed data or Mann–Whitney U test for non-normally distributed data were used for comparison of continuous variables between groups. A p-value of ≤ 0.05 was considered to be statistically significant. Noted range values are minimum–maximum.

## Results

### Validation of mouse adenomyosis model

**Fig. 3:**
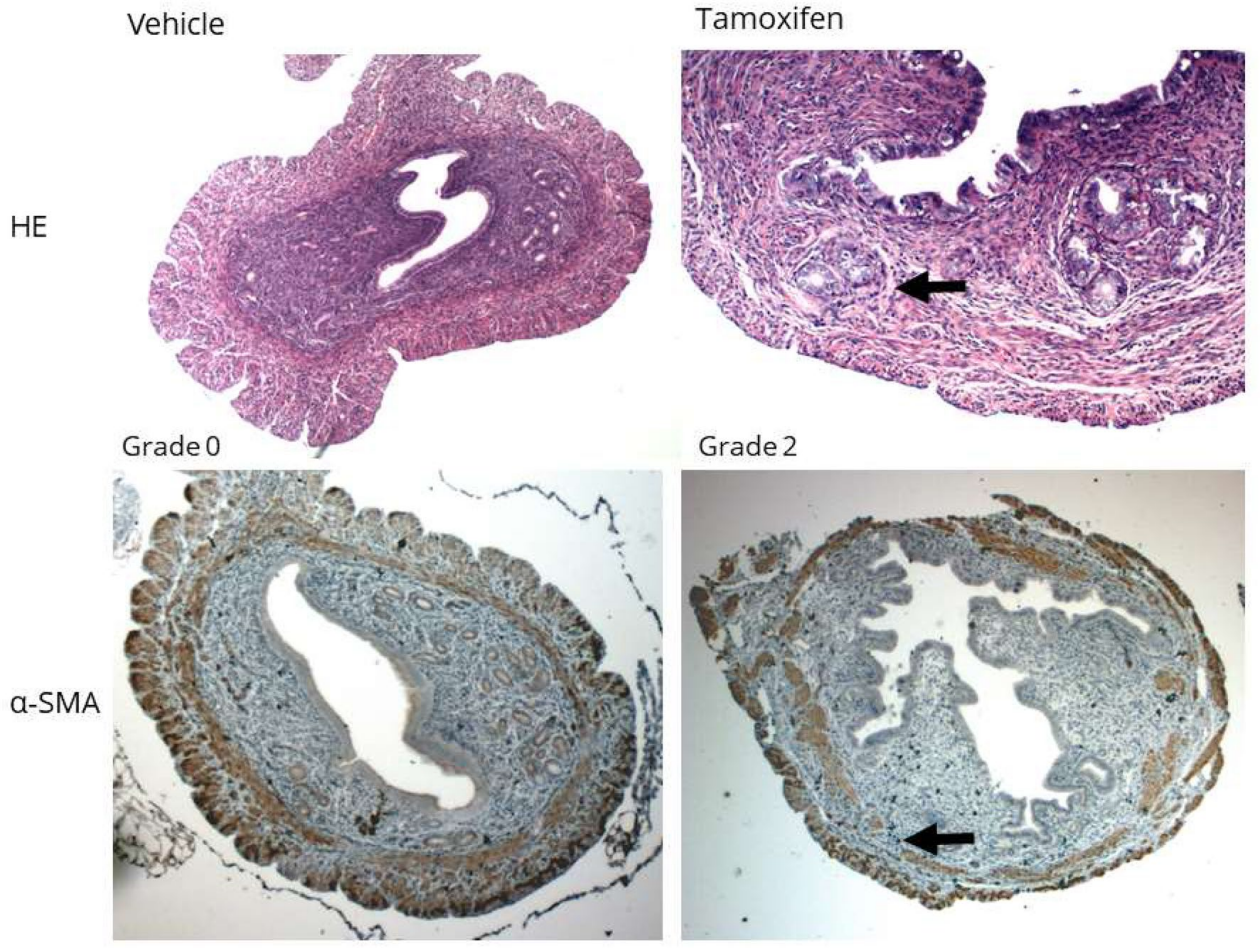
Representative images of HE (top row) and α-SMA (bottom row) stained transverse sections of mice uteri after neonatal treatment with vehicle (left column) and tamoxifen (right column). The uteri of vehicle treated mice show no signs of ectopic endometrium in the myometrium (Grade 0) on the HE stained slice, and an intact myometrium on the α-SMA stained slice, while the uteri of tamoxifen treated mice demonstrate ectopic endometrium (black arrow) in the myometrium (Grade 2 presented) on the HE stained slice, and an interrupted myometrium on the α-SMA stained slice with thin residual unaffected myometrium

To test for the success rate of adenomyosis-induction in mice, 6 female neonatal mice received tamoxifen (daily at day 2–5) and 6 vehicle only. In all mice that received tamoxifen adenomyosis was observed, while none in the vehicle only groups showed the disease (Supplemental Table 1). All mice that received tamoxifen developed adenomyosis defined as grade 2 (Fig. 3). In the vehicle-treated group, a clearly visible junctional zone was present, with a distinct separation between the endometrium and myometrium visible on HE-stained as well as α-SMA stained uteri.

In α-SMA stained uteri, there was loosening and disruption of the circular layer of smooth muscle cells of the inner myometrium in the tamoxifen-treated group, with several glands interrupting and infiltrating the inner myometrium, leading to thin residual unaffected myometrium and hypertrophied adjacent myometrium (Fig. 3). The residual unaffected myometrium in comparison to the total uterine wall was significantly smaller in the tamoxifen-treated compared to the vehicle-treated group (p < 0.05). In addition, the ratio of the thinnest point of the myometrium to the thickest point of the myometrium was smaller in the tamoxifen-treated group (p < 0.05) (Supplemental Table 1). Overall, the results of the α-SMA staining show that the residual unaffected myometrium is thin at the site of an adenomyosis lesion, while increased myometrium thickness is seen adjacent to the affected sites. This may indicate myometrium hypertrophy, a hallmark of adenomyosis. Vimentin expression was observed in endometrial stroma and blood vessel walls, in both tamoxifen and vehicle-treated mice. Staining was also observed in ectopic adenomyotic endometrium stroma of mice treated with tamoxifen. No signs of fibrosis surrounding the adenomyosis affected areas in the tamoxifen-treated group were observed by H&E or PTAH staining. Microvascular density (MVD) was highest in the eutopic endometrium in both the vehicle and tamoxifen-treated groups, without a difference between the groups (Supplemental Table 1). There was no difference in MVD between the ectopic and eutopic endometrium, but the MVD in all types of endometrium was higher than in the myometrium (p < 0.05).

### Treatment outcome

#### Prevalence and severity of adenomyosis

**Fig. 4:**
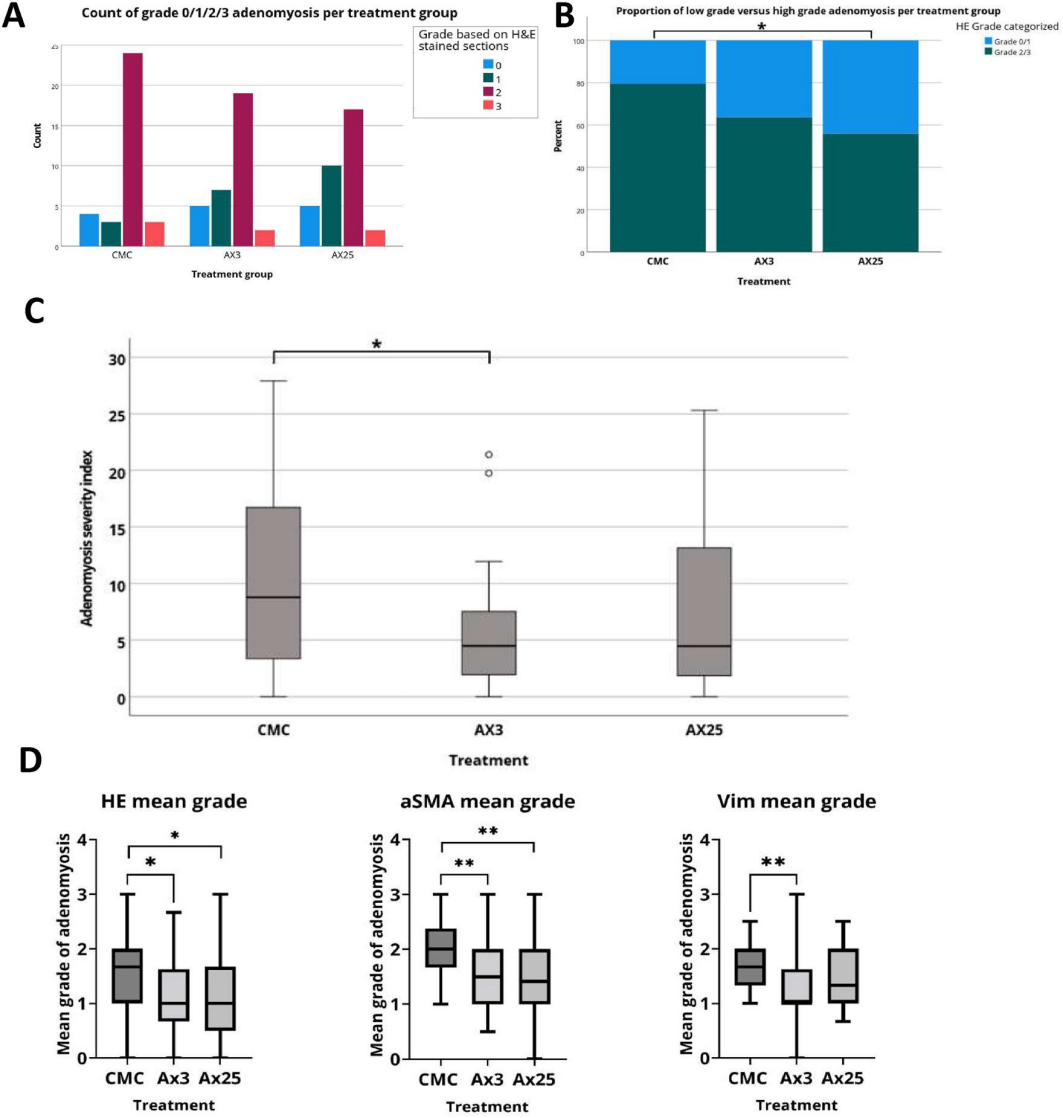
**A** Bar chart of the prevalence (count) of Grade 0/1/2/3 adenomyosis on the HE stained mice uteri, per treatment group. **B**, Proportion of low grade versus high grade adenomyosis per treatment group. **C** Adenomyosis severity score (mean grade * mean percentage surface area of adenomyosis per mouse uterus specimen) per treatment group. Mean grade of adenomyosis of all transverse sections per mouse uterus per treatment group. **D** Mean grade of adenomyosis of all transverse sections per mouse uterus, per staining method and per treatment group. Data are shown as median ± range for all mice pooled. * significance p < 0.05, ** p < 0.001. Treatment groups: placebo (CMC, n = 34), AX3 (axitinib 3 mg/kg, n = 33) and AX25 (axitinib 25 mg/kg, n = 34)

A total of 101 mice completed the neonatal tamoxifen induction and could be analyzed (Table 1). The majority of all mice had developed adenomyosis, without a difference between the treatment groups (p = 0.78, Fig. 4A). The prevalence of high grade (2/3) adenomyosis was significantly lower in mice treated with axitinib dose II (n = 19, 55.9%) than in the placebo group (n = 27, 79.4%, *X*^2^ p < 0.05) (Fig. 4B). The grade of adenomyosis was 0.7 point lower in the groups treated with axitinib dose I (3 mg/kg) and dose II (25 mg/kg) than in the placebo treated group (dose I and II both median of 1.0 compared to 1.7, K-W p < 0.05, post-hoc analyses differences placebo and dose I and II treated groups both p < 0.05). The adenomyosis severity index (mean grade * mean area of adenomyosis) was reduced by 48% in the mice who were treated with axitinib dose I compared to the placebo treated mice (difference between groups K-W = 0.07, post hoc difference between placebo- and AX3 treated group p < 0.05 and between placebo and AX25 treated group p = 0.173) (Fig. 4C). While HE staining was the primary tool to assess the presence of ectopic endometrium glands, α-SMA staining demarcated the location of myometrium interruption and vimentin staining depicted whether this interruption was endometrium stroma. The mean grade of adenomyosis was calculated per staining method and resulted in comparable outcomes (Fig. 4D). There was no significant difference in the number of ectopic glands, or diffuse versus focal spread of adenomyosis between the groups (Table 1).

**Table 1:** Characteristics of mice in anti-angiogenesis treatment experiment

Characteristic	Placebo CMC-only n = 34	Dose I Axitinib 3 mg/kg n = 33	Dose II Axitinib 25 mg/kg n = 34	P-value
Weight end treatment (g) Mean SD	29.9 3.1	28.4 2.2	28.9 3.1	0.07
Adenomyosis (n, %) No Yes	4 30	3 30	5 29	0.78
Grade (n, %) 0 1 2 3	4 (11,8%) 3 (8,8%) 24 (70,6%) 3 (8,8%)	5 (15,1%) 7 (21,2%) 19 (57,6%) 2 (6,1%)	5 (14,7%) 10 (29,4%) 17 (50,0%) 2 (5,9%)	0.50
Ectopic glands, median [Range] Missing	3,3 [0–27] 1	2,7 [0–11] 5	3,0 [0–11] 1	0.8
Spread None Focal Diffuse Missing	4 (11,8%) 11 18,6 0	5 (15,2%) 11 16,4 4	5 (14,7%) 8 18 1	0.8
Microvessel density, mean ± SD Endometrium Ectopic endometrium Myometrium	4.7 ± 1.3 4.1 ± 1.0 2.9 ± 0.6	4.4 ± 1.1 3.8 ± 1.3 2.6 ± 0.5	4.6 ± 0.7 4.0 ± 1.0 2.9 ± 0.70.4	0.609 0.577 0.038
Myometrium parameters, median [range] Residual unaffected/thickest myometrium Adjacent thickest myometrium/uterus wall Adjacent thickest myometrium/uterus diameter	0.19 [0.08–0.65] 0.55 [0.42–0.77] 0.20 [0.10–0.28]	0.23 [0.11–0.55] 0.54 [0.25–0.78] 0.18 [0.10–0.25]	0.26 [0.09–0.71] 0.49 [0.28–0.69] 0.18 [0.11–0.30]	0.055 0.007 0.023

*SD* standard deviation, *En* endometrium, *M* myometrium

### Uterine wall parameters

**Fig. 5:**
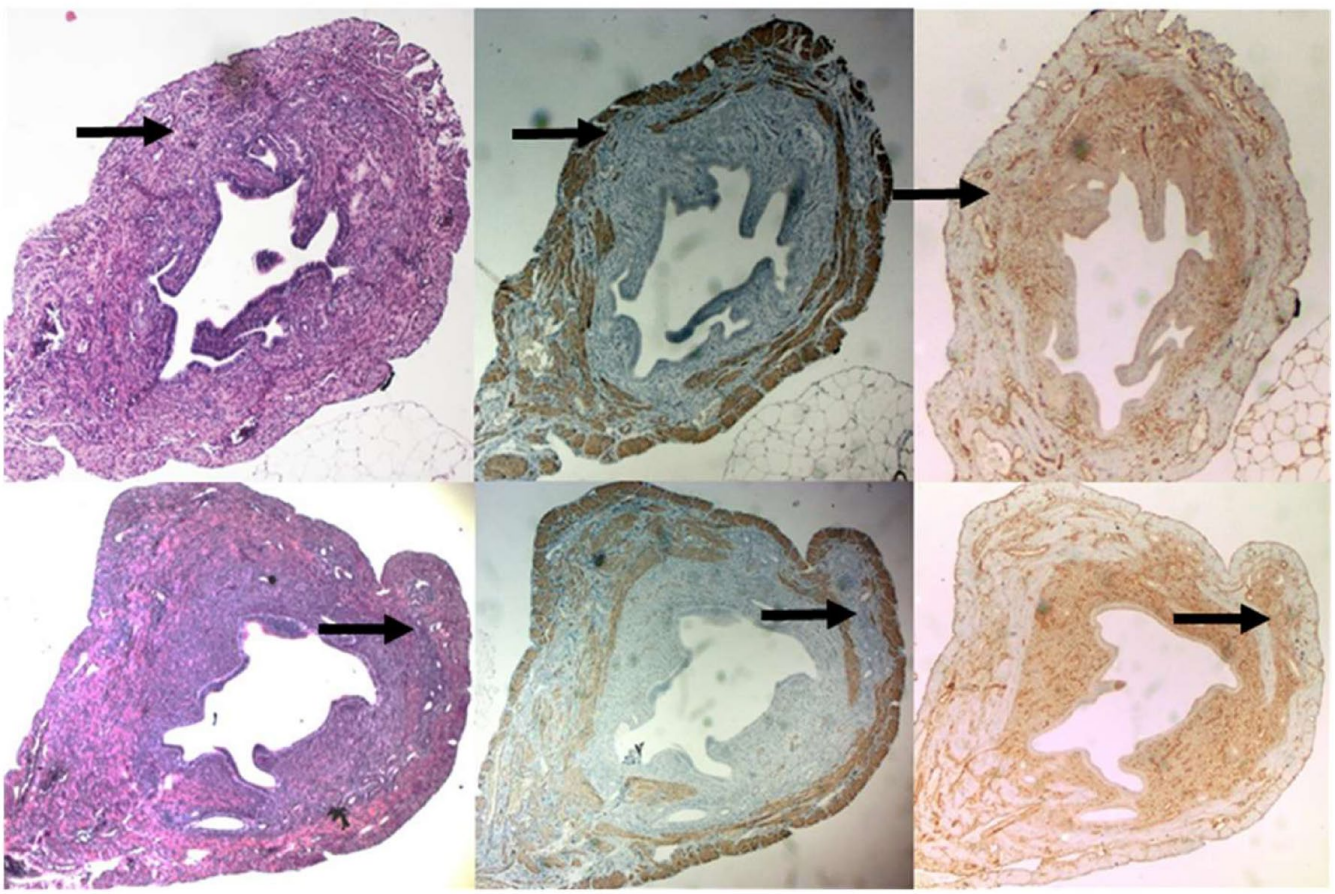
Top: From left to right the HE, α-SMA and Vimentin staining results of grade I adenomyosis in transverse sections of mice uteri. Bottom: from left to right the HE, α-SMA and Vimentin staining results of grade II adenomyosis in transverse sections of mice uteri. Black arrow indicates the invaded ectopic endometrium with thin residual unaffected myometrium in each uterine transverse section

Expression of α-SMA was a clear marker of the longitudinal muscle layer of the myometrium of the mouse uteri. In all tissue sections of both the axitinib as well as placebo-treated mice who had adenomyosis, the junctional zone was interrupted, in accordance with the tamoxifen group of the validation experiment (Fig. 5). The thickest myometrium adjacent to the affected myometrium compared to the uterine wall and to total uterine diameter was larger in the placebo group compared to the axitinib-treated groups (both K-W p < 0.05 with post hoc differences between placebo- and dose I/AX3 and dose II/AX25 treated groups both p < 0.05) (Table 1). Also, the residual unaffected myometrium compared to the adjacent thickest myometrium was smaller in the placebo treated group, although this did not reach significance (K-W p = 0.055).

### Blood vessel parameters

**Fig. 6:**
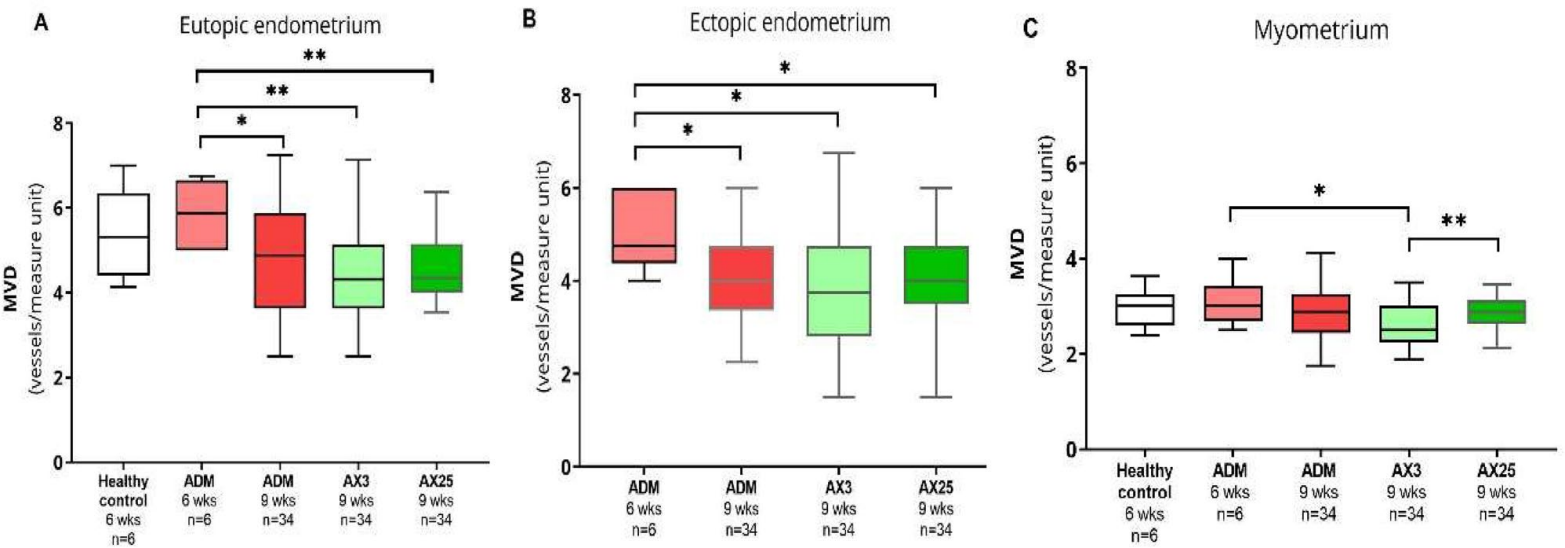
Microvascular density per unit of measurement in the eutopic endometrium (**A**), ectopic endometrium (**B**), and myometrium (**C**) of mice from the validation experiment treated with vehicle (Healthy control n = 6) or tamoxifen (ADM, n = 6) and sacrificed at 6 weeks of age, and the mice with tamoxifen-induced adenomyosis treated with axitinib 25 mg/kg (Ax25, n = 34), axitinib 3 mg/kg (Ax3, n = 34), or placebo (ADM, n = 34) sacrificed at 9 weeks of age. Data shown as median with interquartile range. * significance p < 0.05, ** p < 0.001

Similar as in the validation experiment, microvascular density (MVD) was highest in eutopic endometrium and lowest in the myometrium, in all treatment groups (Table 1, Fig. 6). Although no increase in MVD was seen in tamoxifen-treated mice compared to healthy controls at six weeks, MVD in both eutopic and ectopic endometrium was significantly lower in all mice after placebo or axitinib treatment sacrificed at nine weeks, than in mice sacrificed at 6 weeks after tamoxifen treatment. In the myometrium, there was a significant difference between the treatment groups (K-W p = 0.038). The MVD was lower in the treatment group that received dose I axitinib compared to the dose II group (p = 0.008), the difference between dose I and the placebo treated group did not reach significance (p = 0.055).

#### RT-qPCR

**Fig. 7:**
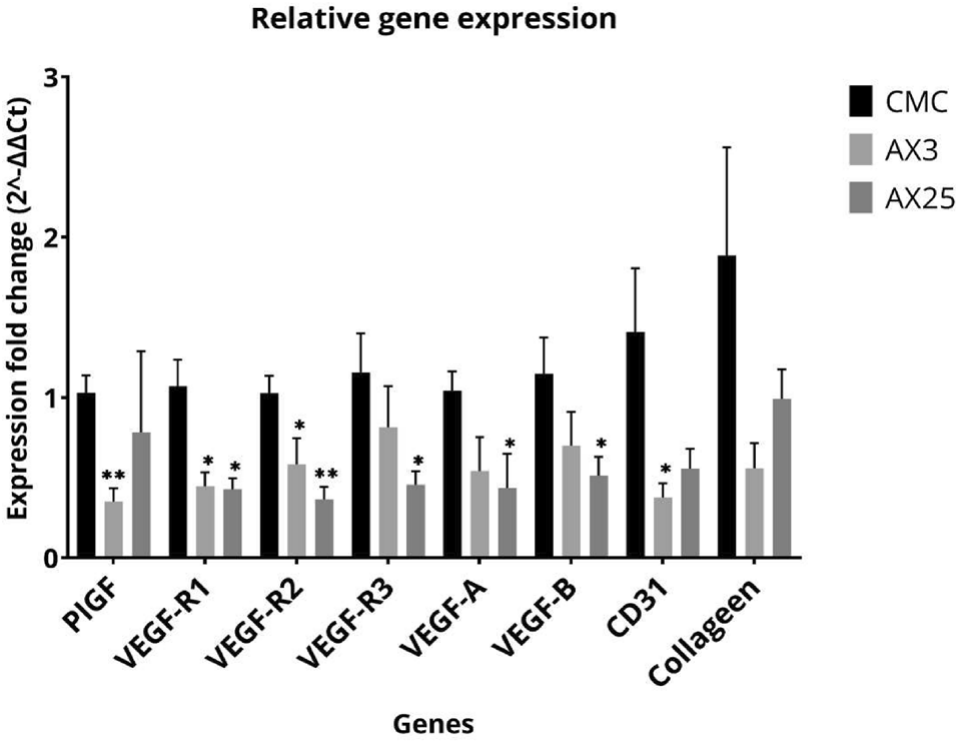
Real-time (RT)-PCR molecular profiling of the mouse uteri treated with CMC, axitinib dose I (AX3 = 3 mg/kg), or axitinib dose II (AX25 = 25 mg/kg). Expression fold change of angiogenesis-related genes plGF, VEGF-R1, VEGF-R2, VEGF-R3, VEGF-A, VEGF-B, CD31 and Collagen determined by 2^- ΔΔCt of RT-PCR. Mean relative expressions are shown as mean with the SEM. CMC, n = 7; AX3, n = 6; AX25, n = 7. *p < 0.05 and **p < 0.001

Real-time quantitative PCR studies were performed in a random selection of for uterus specimens exposed to axitinib (dose I, n = 7; dose II, n = 6) or placebo, n = 7). We investigated the expression of CD31 to confirm the results of the manual MVD count. In addition, we measured expression of angiogenesis related factors, including VEGF-A, VEGF-B, VEGF receptor 1 (VEGF-R1), VEGF-R2, VEGF-R3, fibroblastic growth factor (FGF), and placental growth factor (PlGF). The expression of each gene was normalized to cyclophilinA that had most constant Ct values between the groups (Supplemental Fig. 1). It was observed that treatment with dose I axitinib resulted in a downregulation of VEGF-R1 by 63% (p = 0.003), and of VEGF-R2 by 45% (p = 0.038) (Fig. 7). Treatment with dose II axitinib resulted in downregulation of all VEGF receptors (1–3), as well as VEGF-A and VEGF-B by over 60% (p < 0.05). Also the expression of CD31 decreased over 85% after treatment with dose I or II axitinib (p = 0.04 and p = 0.06).

## Discussion

Angiogenesis inhibition in a murine mouse model of tamoxifen-induced adenomyosis through the administration of axitinib, a VEGF-R2 tyrosine kinase inhibitor, leads to a reduction in the severity of adenomyosis. The prevalence of grade 2/3 adenomyosis was significantly lower in the axitnib dose II (25 mg/kg) group than in the placebo group, the mean grade of adenomyosis was significantly lower in the both axitinib dose I (3 mg/kg) as well as dose II group compared to the placebo group. The adenomyosis severity index was significantly lower in the group treated with axitinib dose I than in the placebo-treated group. Furthermore, in the placebo group the myometrium was thicker adjacent to the affected site than in both axitinib treated groups. Together with the tendency towards a smaller residual unaffected myometrium in the placebo group, these results indicate more severe myometrium infiltration and hypertrophy in the placebo- than in the axitinib-treated groups. The gene expression of VEGF-R1, VEGF-R2, and VEGF-R3, as well as VEGF-A and VEGF-B was markedly reduced after axitinib treatment. Also the expression of CD31 was significantly reduced in the axitinib dose I treated mice, although no significant difference in MVD was found between the axitinib and placebo treated groups. These results support our hypothesis that angiogenesis inhibition is a promising therapeutic approach for adenomyosis. Although treatment with the lower dose axitinib resulted in decreased gene expression of only VEGF-R1 and –R2, the observed effect of dose I on the expression of CD31, the lower MVD compared to dose II in the myometrium, and the significant effect on the mean grade and severity index of adenomyosis, confirm our hypothesis that a lower dose of angiogenesis inhibitor could be effective to treat a benign condition such as adenomyosis.

The notion that angiogenesis inhibition might have beneficial effects in the treatment of adenomyosis has been noted in other preclinical studies. A study by Jin et al.[32] investigated the therapeutic effect of NSAIDs in the same model as was used in the present study and found that both NSAIDs and celecoxib reduced the depth of endometrial infiltration in the myometrium. Celecoxib, which is a selective COX-2 inhibitor, demonstrated the greatest effect and in addition had an analgesic effect by prolonging the thermal response latency. However, only the naproxen treated mice demonstrated a significant reduction in vessel density. While the COX-induced inhibition of angiogenesis has also been investigated in the treatment of cancer, the association between COX-2 inhibition and angiogenesis has not been fully established. The authors of this study suggest that the observed reduction in adenomyosis is due to celecoxib’s effect on the suppression of estrogen production demonstrated by the lower concentration of estrogen and aromatase P450, reversing epithelial-to-mesenchymal transition and reducing uterine fibrosis. Important to note is that Jin et al. started the NSAID treatment at the age of day 6, while it has been described that neonatal tamoxifen exposure leads to adenomyosis at 10 days at the earliest and to fully established adenomyosis by 6 weeks of age [33]. So, starting treatment before that time might prevent rather than treat adenomyosis in mice. Zhu et al. [31] also used the tamoxifen-induced mice model, but used it to test Ozagrel treatment, which is an anti-mouse GPIbα polyclonal IgG antibody that depletes platelets, from week 16 of age. They found that platelet depletion led to suppressed myometrial infiltration, improved hyperalgesia, reduced uterine contractility, lowered plasma corticosterone levels and slowed down fibrogenesis. Platelet activation is related to angiogenesis since its co-occurrence with TGF-β1 release leads to EMT and fibroblast-to-myofibroblast transdifferentiation. Although these results are promising and point towards the involvement of increased platelet aggregation in adenomyosis, the clinical application of anti-platelet therapy is limited due to the risk of hemorrhage to occur.

Other studies have also aimed to target angiogenesis in different murine adenomyosis models. Zhou et al.[35] implanted a single pituitary gland in SHN mice at 7 weeks of age, to induce adenomyosis through hyperprolactinemia [36]. Treatment started one day after pituitary grafting until week 13 and was aimed at inhibiting angiogenesis by administration of TNP-470, a fumagillin analogue that inhibits endothelial cell migration and proliferation. The TNP-470-treated group did not develop signs of uterine adenomyosis as opposed to 80% in the control group, and their mean surface area of blood vessels in the endometrium reduced to 60.5% of that in the control group. Although these results were promising, no further research has been reported on the use of TNP-470 for this application. Huang et al.[15] found that treatment with bevacizumab (anti-VEGF monoclonal antibody), administered 14 days after ovariectomized mice were xenografted with human adenomyosis lesions at week eight, decreased the MVD and the expression of VEGF. In a similar model, Zhou et al. [37], demonstrated that knockdown of annexin A2, an estrogen-responsive protein, reduced angiogenesis, suggesting its potential as therapeutic target. However, both studies using the xenotransplantation method are incomparable to the present study, since the adenomyosis was xenotransplanted intraperitoneally, and thereby becomes endometriosis. Neither study evaluated the effect on adenomyosis in utero.

### Strengths and limitations

The strength of the present study is the optimal study design, in which the most accurate adenomyosis animal model was chosen and validated before starting the experiment. Also, an appropriate sample size was used in order to reach relevant and reliable study outcomes. Since treatment was started only after adenomyosis was certainly induced, this study represents outcomes that are eventually applicable in a clinical setting, where women will seek treatment for adenomyosis only after the diagnosis has been established. This way, a full eradication of the adenomyosis would probably not have been feasible, but since this study was powered to evaluate a clinical relevant reduction of adenomyosis instead, the present results are promising and reliable. Another strength is the elaborate assessment of the presence and severity of adenomyosis based on three complimentary histological methods that was applied in this study. Other studies defined the severity merely on the grade of adenomyosis, without taking the affected surface area, or degree of affected myometrium into account. Although there is no existing guideline on the evaluation of the severity of adenomyosis, the approach that was used in this study, seems more reliable since it takes both the depth and surface area of ectopic endometrium infiltration as well as the affected myometrium into account. Limitations of this study include the uncertainty on the optimal timing to start treatment, and the assessment of the presence and severity of adenomyosis. First, the optimal timing to start treatment is difficult to establish since it is unknown exactly when angiogenesis is most crucial in the establishment of endometrium at ectopic sites, both in mice and human. Theoretically, in humans each menstrual cycle induces tissue injury and repair, which together with elevated paracrine estrogen exposure, might lead to invagination of ectopic endometrium [38, 39]. This establishment of endometrium at ectopic sites is accompanied by angiogenesis, but in a further stage, might also lead to fibrosis [40]. Although no signs of uterus fibrosis were observed in the present study, the establishment of ectopic endometrium might have been in a relatively progressed stage. Possibly, this explains why the MVD was higher in the endometrium of the mice in the validation experiment that were sacrificed at 6 weeks compared to the MVD in the endometrium of mice sacrificed at 9 weeks. Therefore, future studies should test different start of treatment timings and perhaps aim to start anti-angiogenesis therapy at an earlier stage. Another limitations is that we did not study into detail which form of angiogenesis was most prevalent. Angiogenesis is often thought to directly correlate with an increase in MVD. However, other forms of angiogenesis such as coalescent angiogenesis or vessel elongation would not necessarily increase the MVD [41]. Illustrative is the finding in this study that there was a difference gene expression of CD31 between the placebo and axitinib-treated groups, which was not evident from the MVD evaluation. Also, the occurrence of vessel elongation might explain the observed reduced MVD in the placebo treated group sacrificed at 9 weeks versus the tamoxifen-only treated group sacrificed at 6 weeks. To gain more understanding of the angiogenic process underlying adenomyosis, the different forms of angiogenesis should be further explored.

### Future perspectives

Since there is currently no treatment option for premenopausal women who wish to retain their uterus and their fertility, treatment with an angiogenesis inhibitor might provide a solution. Although we tested axinitib in this study, we are aware that this is a potent drug for a.o. advanced kidney cancer, and can have serious side-effects that are accepted when treating a life-threatening disease. The major requirement in further development of anti-angiogenesis therapy for adenomyosis is a safety and side-effect profile tolerable for pre-menopausal women. Since angiogenesis has an important function in ovarian function and regeneration in the menstrual cycle, the application would need to be selective so that physiological functions are preserved. Therefore, future research should include assessment of different types of angiogenesis inhibitors, as well as testing the lowest possible effective, locally applied dose. Our mice did show reduced severity of adenomyosis using axitinib at the lower of the two doses, yet future research is required to evaluate potential side effects at low-dose application.

**In conclusion,** angiogenesis inhibitor axitinib successfully reduced the severity of adenomyosis by approximately 50%. These are promising first results for the treatment of adenomyosis, but further research should focus on commonality among different angiostatic drugs, along with potential side effects of low-dosage, the method and timing of application.

## Supplementary Information

Below is the link to the electronic supplementary material.

Supplementary file1 (DOCX 258 KB)

## Data Availability

The data that support the findings of this study are available on request from the corresponding author, MJH.
