# Supplementary material for: Anti-angiogenic therapy as potential treatment for adenomyosis
Source: Angiogenesis. 2025 Jan 25;28(1):12. doi: 10.1007/s10456-024-09960-6 (PMC11762773; doi:10.1007/s10456-024-09960-6)
Supplement: Supplementary file 1 — Supplementary file1 (DOCX 258 KB) [file 10456_2024_9960_MOESM1_ESM.docx]

| Supplemental Table 1 Characteristics of mice in validation experiment | | | |
| --- | --- | --- | --- |
| Characteristic | **Vehicle**  **(n=6)** | **Tamoxifen**  **(n=6)** | **P-value** |
| Weight  Median  Range | 25.5  22.7 – 28.8 | 25.2  23.1 – 29.8 | 0.936 |
| Adenomyosis  No  Yes | 6 (100%)  0 (0%) | 0 (0%)  6 (100%) |  |
| Grade  0  1  2  3 | 6 (100%)  0 (0%)  0 (0%)  0 (0%) | 0 (0%)  0 (0%)  6 (100%)  0 (0%) |  |
| Ectopic glands  Median  Range | 0  0 | 7.5  3 - 15 |  |
| Spread  Focal  Diffuse | 0 (0%)  0 (0%) | 2 (33.3%)  4 (66.7%) |  |
| Microvessel density, median [range]  Endometrium  Ectopic endometrium  Myometrium | 5.3 [4.1-7.0]  -  3.0 [2.4-3.6] | 5.9 [5.0-6.8]  4.8 [4.0-6.0]  3.0 [2.5-4.0] | 0.420  -  0.699 |
| Myometrium parameters (α-SMA), median  Residual unaffected/thickest myometrium  Residual unaffected myometrium/uterine wall  Adjacent thickest myometrium/uterine wall | 0.65  0.58  0.40 | 0.19  0.18  0.43 | 0.004  0.004  1.000 |
| *Abbreviations: SD, standard deviation; En, endometrium; M, myometrium.* | | | |


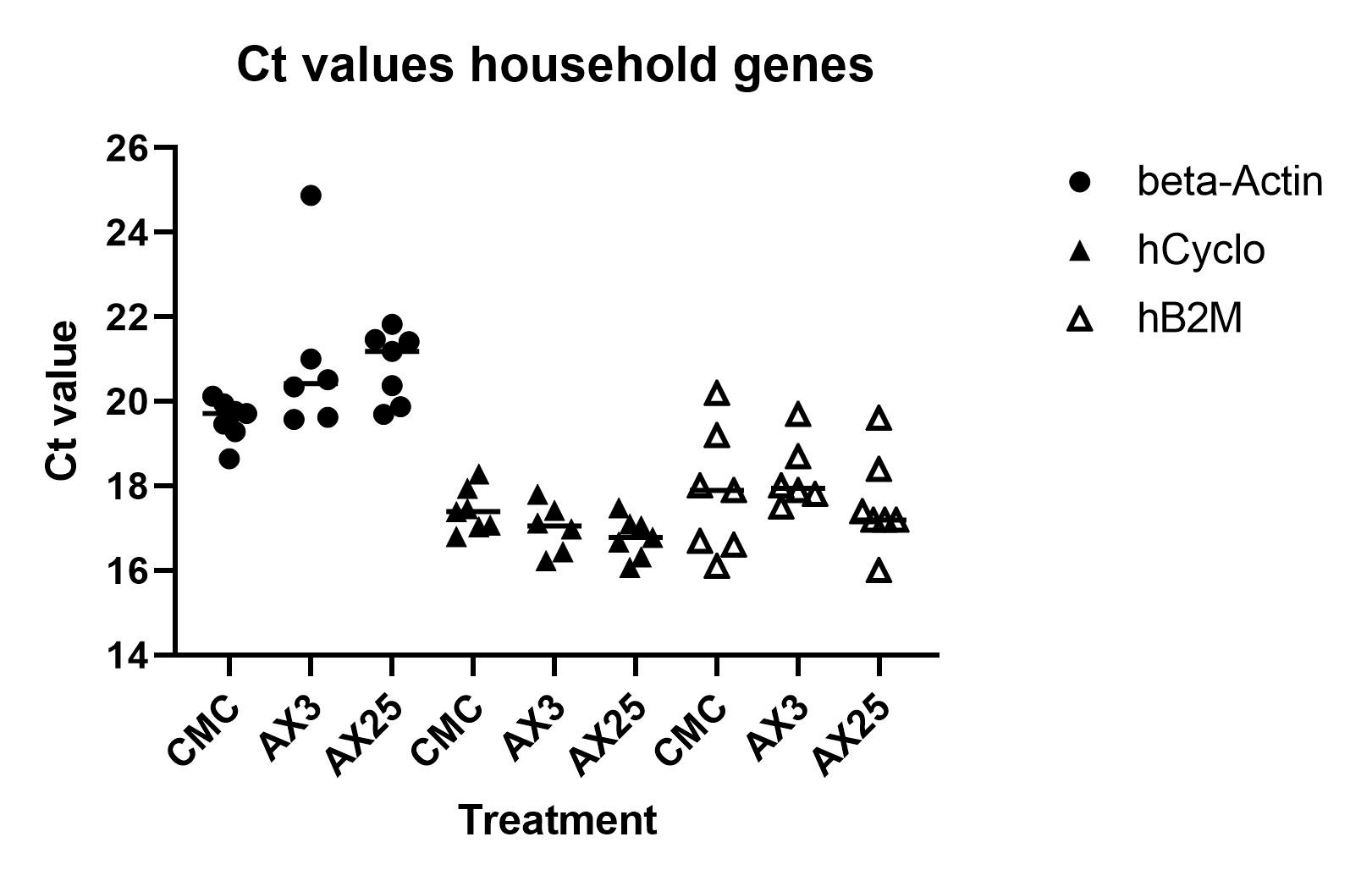


***Supplemental Figure 1****. Ct values of all household genes between the groups, showing that cyclophilinA had most constant Ct values between the groups*
